# A novel computational method to predict hypoattenuated leaflet thickening post-transcatheter aortic valve replacement using preprocedural computed tomography scans

**DOI:** 10.1016/j.xjse.2024.100041

**Published:** 2024-12-24

**Authors:** Aniket Venkatesh, Fateme Esmailie, Noah Tregobov, Hoda Hatoum, Breandan Yeats, Huang Chen, Beom Jun Lee, Philipp Ruile, Franz-Josef Neumann, Philipp Blanke, Jonathon Leipsic, Gaurav Gulsin, Vinod Thourani, David Meier, Lakshmi Prasad Dasi, Stephanie L. Sellers

**Affiliations:** a Parker H. Petit Institute for Bioengineering and Bioscience, Georgia Institute of Technology, Atlanta, Ga; b Wallace H. Coulter Department of Biomedical Engineering, Georgia Institute of Technology, Atlanta, Ga; c Department of Biomedical Engineering, University of North Texas, Denton, Tex; d Cardiovascular Translational Laboratory, St. Paul's Hospital and University of British Columbia, Vancouver, Canada; e Department of Radiology, St. Paul's Hospital and University of British Columbia, Vancouver, Canada; f Department of Biomedical Engineering, Michigan Technological University, Houghton, Mich; g Department of Biomedical Engineering, University of Nevada, Las Vegas, Las Vegas, Nev; h Department of Cardiology and Angiology, Medical Center, University of Freiburg, Faculty of Medicine, Bad Krozingen, Germany; i Department of Cardiovascular Sciences, University of Leicester and the NIHR Leicester Biomedical Research Centre, Glenfield Hospital, Leicester, United Kingdom; j Department of Cardiovascular Surgery, Marcus Heart Valve Center, Piedmont Heart Institute, Atlanta, Ga; k Department of Cardiology, Lausanne University Hospital and University of Lausanne, Lausanne, Switzerland

**Keywords:** transcatheter aortic valve implantation/replacement, thrombosis, hypoattenuated leaflet thickening, machine learning, computed tomography, reduced order modeling

## Abstract

**Objective:**

Hypoattenuated leaflet thickening (HALT) is a computed tomography (CT) finding after transcatheter aortic valve replacement (TAVR) that is indicative of bioprosthetic valvular thrombosis. There are currently no standardized or validated methods for predicting HALT, which can cause bioprosthetic valve dysfunction and has been associated with adverse patient outcomes. The objective was to develop a novel fast-response, artificial intelligence, and machine learning (ML)-driven computational pipeline to predict HALT using preprocedural CT scans.

**Methods:**

The pipeline consisted of (1) pre-TAVR CT reconstruction and reduced order modeling simulations to automatically predict postprocedural geometric parameters, (2) a landmark-guided automated left ventricle segmentation method to predict hemodynamic parameters, and (3) statistical and ML analyses to develop HALT predictive metrics.

**Results:**

Pre- and postprocedural scans from 45 patients (21 with HALT, 24 without) were used as inputs for the pipeline. We identified statistically significant relationships between HALT and peak systolic blood velocity (*P* < .01) and peak systolic blood flow through the bioprosthetic valve (*P* < .01), left ventricular ejection time (*P* < .01), ejection volume (*P* < .05), and right coronary height (*P* < .05). ML-yielded metrics related to circulation in the neosinuses correlated strongly with HALT occurrence (*P* < .001) along with the greatest accuracy of 84.40% and area under receiver operating characteristic curve of 0.87.

**Conclusions:**

A computational pipeline using pre-procedural CT scans as inputs that outputs post-TAVR geometric and hemodynamic measurements was developed to assess metrics with the potential to predict the risk of HALT. Such a tool may help guide decision-making and understanding of prevention of postprocedural thrombosis.

## Graphical Abstract

**Figure:**
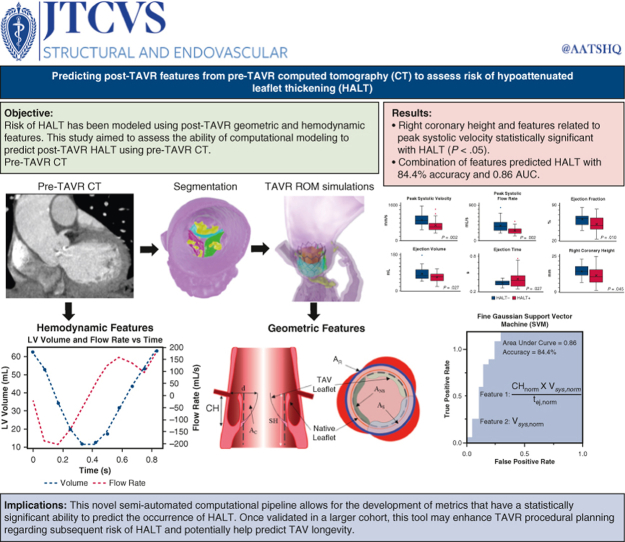

**Figure:**
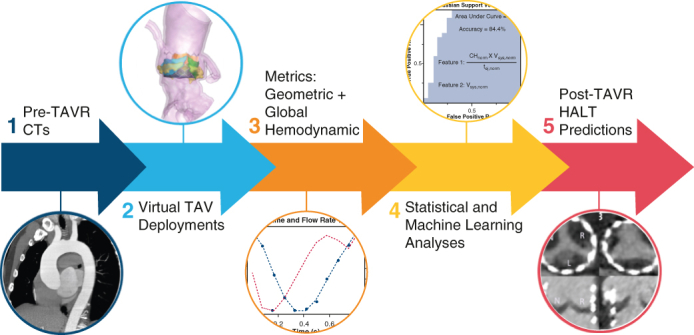
Computational pipeline that uses pre-TAVR CT to develop post-TAVR HALT predictive metrics.

**Central Message**

Computational modeling can complement TAVR procedural parameters in identifying and evaluating HALT predictive metrics from pre-TAVR CT. Testing will require larger cohort and patient parameters.

**Perspective**

Currently, there are no validated methods to predict post-TAVR HALT risk. In this work, a computational modeling pipeline was developed using patient-specific pre-TAVR CTs to generate metrics that were predictive of HALT. Subsequent stages of refinement and validation may yield a pipeline that can identify those at increased risk of HALT and possibly facilitate risk stratification.

Hypoattenuated leaflet thickening (HALT) is a computed tomography (CT) finding after transcatheter aortic valve replacement (TAVR)^1^, ^2^, ^3^, ^4^ that occurs in approximately 10% to 15% of patients.^5^, ^6^, ^7^, ^8^ HALT refers to curvilinear meniscoid thickening extending from the base of a bioprosthetic leaflet that can occur within days of implantation and often represents thrombus.^5^ HALT is a dynamic finding that can appear and resolve at variable times postimplant^3^ but has implications for valve function, durability, and patient symptoms. HALT has been associated with increased transvalvular gradients,^5^ reduced leaflet motion,^5^ and valve degeneration.^9^ Thus, there is a need for improved patient risk stratification to guide monitoring for HALT and associated adverse outcomes post-TAVR.

Factors such as patient anatomy,^10^ implantation parameters including degree of expansion^10^ and height of implantation,^11^ blood type,^11^ hemodynamic characteristics,^11^ and medication history^12^^,^^13^ have been associated with HALT development. However, predicting those who will develop HALT is a complex problem. HALT prediction models are developed on the basis of data-informed methods^4^^,^^14^^,^^15^ or physics-informed methods.^10^ Data-informed methods include analysis of post-TAVR computed tomography (CT) scans of patients with HALT.^3^^,^^4^^,^^6^^,^^8^ However, conclusions drawn from use of data-informed methods are limited, as they often cannot be generalized to different valve types and patient populations. In contrast, physics-informed computational methods are determined by application of computational fluid dynamic (CFD),^14^, ^15^, ^16^, ^17^ fluid structure interaction (FSI),^14^^,^^18^^,^^19^ artificial intelligence,^20^^,^^21^ and in vitro experimental studies.^22^ It is possible to generalize the results of physics-informed methods. However, CFD and FSI methods are computationally time-consuming and expensive,^14^^,^^15^^,^^23^ making them highly impractical to use for patient-specific pre-TAVR HALT prediction.

Given these challenges, there is currently no standardized and validated method to predict HALT post-TAVR. Therefore, the overarching aim of this work was to develop a novel artificial intelligence and machine learning−driven computational pipeline that can rapidly predict HALT using preprocedural CT scans.

## Methods

### Study Design and Setting

A retrospective study design was applied. The study was a collaboration between Georgia Institute of Technology (Atlanta, Georgia), Freiburg University Hospital (Bad-Krozingen, Germany), and St Paul's Hospital (Vancouver, British Columbia, Canada). Georgia Tech's research board (institutional review board [IRB]) approved the protocol for collaboration with Freiburg University Hospital (H17233), originally on June 2, 2017. The Providence Health Care Research Ethics Board (H21-01544) approved the original study protocol on June 10, 2021, for patients from St Paul's Hospital. Patient written consent for the publication of the study data was waived by both IRBs because of the retrospective nature of the study.

### Patient Population

Pre-TAVR and post-TAVR CT scans of 45 patients who underwent a SAPIEN 3 (Edwards Lifesciences) balloon-expandable valve implantation at Freiburg University Hospital were obtained. These patients were selected from a cohort^24^ made available under IRB protocols. A detailed description of the patient selection criteria, image acquisition, and HALT identification for this cohort is available in a previous study.^24^ In brief, all patients had tricuspid aortic valves, no previous prosthetic valve deployments, and were administered peri- and post-interventional antiplatelet treatment consisting of aspirin or aspirin plus clopidogrel. HALT^+^ or HALT^−^ identification was performed by 2 independent reviewers on the basis of visual inspection of hypoattenuation of the leaflets’ base during the diastolic phase on the CT. Timing of the post-TAVR CT ranged from 4 to 90 days postimplant, and HALT was identified on CT. In total, 24 patients were classified as HALT^−^ and the remaining 21 were classified as HALT^+^.

To assess the accuracies of post-reduced order modeling (ROM) geometric measurements using manual post-TAVR CT measurements, retrospective post-TAVR CT scans of 10 consecutive patients who underwent a SAPIEN 3 valve implantation at St Paul's Hospital were obtained under IRB protocols. Inclusion criteria for these patients included balloon-expandable TAVR implantation with available pre- and postprocedural CT TAVR imaging. At the time of discharge, patients were on an antiplatelet and/or anticoagulation therapy. Exclusion criteria included self-expanding valves or the presence of atrial fibrillation during pre-TAVR CT acquisition. To evaluate the semiautomated left ventricle (LV) blood volume segmentation pipeline against manual segmentation, pre-TAVR CT scans from 8 patients at Freiburg University Hospital were analyzed. These 8 patients were chosen because manual segmentation had already been completed with meticulous postprocessing, ensuring accurate measurements of LV blood volumes for reliable comparison.

### Geometric Measurements Using TAVR Reduced Order Modeling

**Figure 1:**
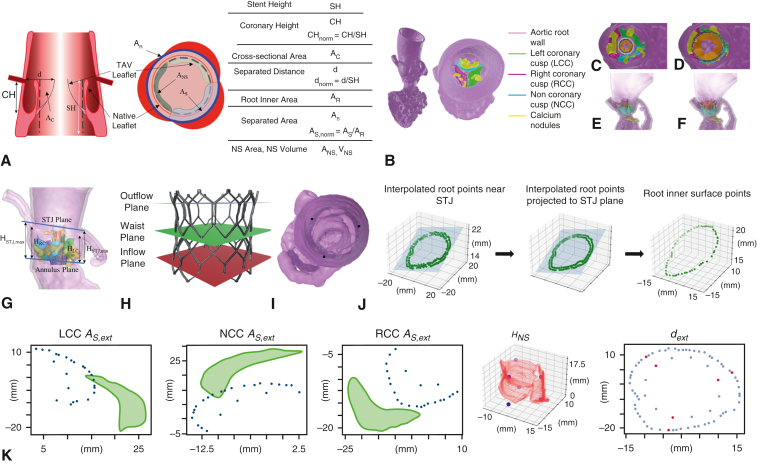
A, Pipeline for semiautomatic post-TAVR geometric measurements. 3D aortic anatomies after manual segmentation of pre-TAVR computed tomography images (B), top (C, D) and side (E, F) views of aortic anatomies before and after expansion of TAV using range of motion, schematic of *H*_*LC*_, *H*_*RC*_, *H*_*STJ,min*_, and *H*_*STJ,max*_ (G), stent planes (H) and regions on aortic root wall (I) along which landmarks were manually selected, steps to obtain root inner surface points (J), and automatically measured stent to root inner wall flow separated areas (*A*_*S,ext*_) along the left coronary cusp (LCC), non-coronary cusp (NCC), right coronary cusp (RCC), neosinus heights (*H*_NS_), and flow separated distances (*d*_ext_) (K). *TAVR*, Transcatheter aortic valve implantation; *3D*, 3-dimensional; *TAV*, transcatheter aortic valve; *H*_*LC*_, left coronary height; *H*_*RC*_, right coronary height; *H*_*STJ,min*_, minimum sinotubular junction height; *H*_*STJ,max*_, maximum sinotubular junction height.

Post-TAVR geometric parameters, such as the neosinus flow-separated area (*A*_S_) and neosinus height (*H*_NS_) measured postprocedurally in previous works^25^ were instead measured from preprocedural CT in this study using a novel semiautomated computational pipeline (see Figure 1, *A*, for all measured geometric parameters). Each patient's aortic root wall, native aortic leaflets, and calcium nodules were segmented and meshed using pre-TAVR CT images in Materialise Mimics (Materialise) (Figure 1, *B*). The segmented aortic anatomies were implanted and expanded with the SAPIEN 3 balloon-expandable transcatheter aortic valve (TAV) using an artificial intelligence−based physics-driven ROM entitled PrecisionTAVI (DASI Simulations Software) (Figure 1, *C-F*, and Video 1). The TAV size, degree of oversizing, depth of implantation, and prosthetic valve commissural alignment were determined in accordance with the post-TAVR CT of each patient before the simulations.

After the ROM simulation, the left and right coronary heights (*H*_LC_ and *H*_RC_, respectively) and the minimum and maximum sinotubular junction (STJ) heights (*H*_STJ,min_ and *H*_STJ,max_, respectively) were manually measured from the deformed aortic root model (Figure 1, *G*). Landmarks along stents of each size were automatically detected along the planes in Figure 1, *H*, whereas landmarks along the STJ plane of the patient-specific deformed aortic roots were manually selected (Figure 1, *I*). The stent landmarks were used to compute the cross-sectional areas along the stent inflow (*IA*), waist (*WA*), and outflow (*OA*) planes using the Shoelace formula:

(1) $$\begin{array}{c} Area=\frac{1}{2}\left(\;\left|\begin{array}{cc} x_{1} & x_{2}\\ y_{1} & y_{2} \end{array}\right|+\left|\begin{array}{cc} x_{2} & x_{3}\\ y_{2} & y_{3} \end{array}\right|+\ldots +\left|\begin{array}{cc} x_{n} & x_{1}\\ y_{n} & y_{1} \end{array}\right|\;\right) \end{array}$$

where *x*_*n*_ and *y*_*n*_ denote the final x and y coordinate values, respectively. These areas were used to calculate the prosthesis deformation index (PDI), a TAV frame factor correlated to frequency of HALT occurrence^10^:

(2) $$\begin{array}{c} PDI=\frac{IA+OA}{2*WA} \end{array}$$

The stent landmarks were also used to calculate the minimum and maximum stent heights (*H*_stent,min_ and *H*_stent,max_, respectively) by finding the difference between the *IA* and *OA* plane heights. The aortic root landmarks were used to isolate the inner wall points at the STJ plane to compute the root area (Figure 1, *J*). An algorithm was developed to automatically detect points along the root inner wall and post-ROM stent and native AV leaflets to measure the stent to root inner wall flow separated area (*A*_S,ext_), flow separated distance (*d*_ext_), and *H*_NS_ for each cusp (Figure 1, *K*). In addition, the stent heights and *d*_ext_ were normalized by the stent sizes, the coronary and STJ heights and *H*_NS_ were normalized by *H*_stent,max_, and each *A*_S,ext_ were normalized by the root area.

High-speed imaging of a 23-mm SAPIEN 3 TAV throughout a cardiac cycle, performed using a pulse-duplicating left heart simulator flow loop,^26^ was used to measure the neosinus opening area (*A*_NS_), TAV leaflet to stent flow separated area (*A*_S,int_), flow separated distance (*d*_*int*_), and the neosinus width (*w*). These measurements were linearly extrapolated for the 20 mm, 26 mm, and 29 mm TAVs. The resulting values are tabulated in Table E1 for all SAPIEN 3 TAV sizes. These parameters were used to compute the neosinus longitudinal cross-sectional area (*A*_C_), total flow separated area (*A*_S_), and total flow separated distance (*d*) for each cusp as follows:

(3) $$\begin{array}{c} A_{C}=\frac{H_{\text{NS}}*w}{2} \end{array}$$

(4) $$\begin{array}{c} A_{S}=A_{S,\text{ext}}+A_{S,\mathrm{int}} \end{array}$$

(5) $$\begin{array}{c} d=d_{\text{ext}}+d_{\mathrm{int}} \end{array}$$

### Hemodynamic Measurements Using Semiautomatic LV Segmentation

**Figure 2:**
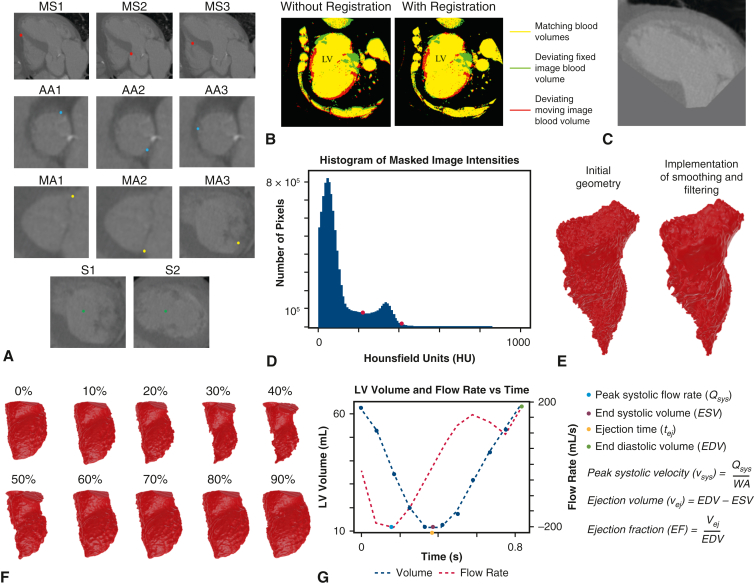
Pipeline for semiautomatic hemodynamic measurements. Landmarks manually selected from the pre-TAVR computed tomography (A), comparison of fixed and moving images without and with registration (B), mask isolating the ROI from remaining anatomies (C), automatic detection of ROI pixel intensity thresholds (D), 3D LV blood volume geometry before and after smoothing and filtering (E), 3D LV blood volume geometries throughout a cardiac cycle (F), and automatically computed hemodynamic measurements (G). *TAVR*, Transcatheter aortic valve implantation; *ROI*, region of interest; *3D*, 3-dimensional; *LV*, left ventricle.

The hemodynamic parameters were determined by a landmark-guided, intensity-based LV segmentation method using patient-specific pre-TAVR CT. This segmentation method used manual landmark selections along the membranous septum (MS1, MS2, MS3), aortic annulus (AA1, AA2, AA3), mitral annulus (MA1, MA2, MA3), and within the LV blood volume (S1, S2) to define the region of interest (ROI) containing the LV blood volume (Figure 2, *A*). These landmarks were used to automatically map the points from CT image of 1 cardiac phase, defined as the fixed image, to the others, defined as moving images, to minimize the impact of LV movement throughout the cardiac cycle (Figure 2, *B*). The fixed image and selected landmarks were used to generate a mask isolating the ROI from the remaining anatomies, which was applied to the moving images (Figure 2, *C*). A histogram of pixel intensities was plotted using the masked image, which was used to automatically compute the minimum and maximum intensity thresholds that capture the LV blood volume (Figure 2, *D*). The 3-dimensional geometries of the LV blood volumes throughout a cardiac cycle were segmented using a marching cubes algorithm and postprocessed using smoothing and filtering algorithms (Figure 2, *E* and *F*).

The cardiac phase percentage was correlated with time points using the patient heart rate extracted from the pre-TAVR CT metadata with the following equation:

(6) $$\begin{array}{c} time\;\left[s\right]=\frac{phase\;\left[\%\right]}{100\%}*\frac{60\;\left[\frac{s}{\min}\right]}{heart\;rate\;\left[\text{bpm}\right]} \end{array}$$

The LV blood volumes were automatically measured plotted with respect to time and a polynomial curve was fitted to the data. The flow rate of blood through the LV was calculated from the derivative of the fitted polynomial at every millisecond throughout the duration of the patient cardiac cycle. The absolute minimum value of LV blood volume indicated the end-systolic volume (ESV) and the absolute maximum value was considered as end-diastolic volume (EDV). Ejection volume (*V*_ej_) was calculated as the difference between the EDV and ESV and represents the stroke volume and normalized by the EDV to give the ejection fraction. Ejection time (*t*_ej_) was approximated as the time to reach ESV. The absolute minimum point in the plot of flow rate versus time was defined as the peak systolic flow rate (*Q*_sys_), which was normalized by the average flow rate (*Q*_avg_). Finally, the peak systolic velocity (*v*_sys_) was calculated by dividing the peak systolic flow rate by the approximate stent cross-sectional area at the waist, as discussed during the calculation of PDI. Like the peak systolic flow rate, *v*_sys_ was normalized by the average velocity. Figure 2, *G*, shows the LV volume and flow rate versus time plots along with the detected hemodynamic measurements.

### Evaluation of Measurements

**Figure E1:**
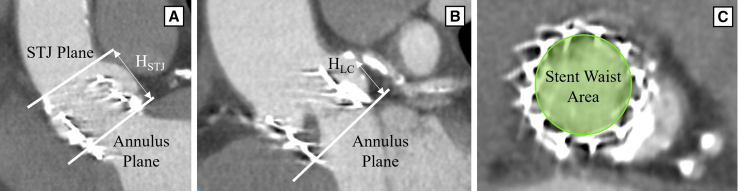
Measurement of post-computed tomography geometric parameters for validation of computational pipeline. Identification of STJ and annulus planes and measurement of *H*_*STJ*_ (A), measurement of coronary heights including HLC (B), and measurement of stent cross-sectional areas including waist area for calculation of PDI (C). *STJ*, Sinotubular junction height; *H*_*STJ*_, sinotubular junction height; *H*_*LC*_, left coronary height; *PDI*, prosthesis deformation index.

Patients’ CT images with *H*_LC_*, H*_RC_*, H*_STJ_, and PDI measured by staff radiologists using post-TAVR CT images (Figure E1) were used to compare the same measurements obtained using the computational pipeline. The mean absolute error and the mean percentage error were calculated for each parameter as follows:

(7) $$\begin{array}{c} MAE=\frac{\sum_{i=1}^{N}\left|P_{2i}-P_{1i}\right|}{N} \end{array}$$

(8) $$\begin{array}{c} MPE=\frac{\sum_{i=1}^{N}\left(\frac{P_{2i}-P_{1i}}{P_{2i}}\right)*100\%}{N} \end{array}$$

where *P*_2_ is the measurement using post-ROM models, *P*_1_ is the measurement using post-TAVR CT, and *N* is the 10 total CTs used.

To perform validation of the LV blood volume segmentation method, the LV blood volumes throughout the cardiac cycle were manually segmented in Materialise Mimics using patient-specific pre-TAVR CT images. The geometries with the smallest and largest volumes, representing the ESV and EDV, respectively, were postprocessed using 10 iterations of the smoothing and filtering algorithms discussed previously. The ESV and EDV were then remeasured using the smoothed meshes. Using Equations 7 and 8, the mean absolute error and mean percentage error were calculated for each measurement.

### Statistical and ML Analyses of HALT Prediction

For each geometric and hemodynamic parameter, a 2-sample *t* test assuming unequal variances was performed to determine whether there was a significant difference in the means of the HALT^+^ and HALT^−^ samples. Parameters that indicated statistical significance (*P* < .05) were used to construct metrics that shared the same dimensions as clinically relevant metrics such as shear stress and blood residence time. These metrics and individual parameters were then used as predictor features and to train various binary classification ML algorithms to classify the patients in 2 classes: HALT^+^ and HALT^−^. The binary classifiers applied in this study included decision trees, support vector machines), logistic regression, naïve Bayes, nearest neighbors, kernel approximation, ensemble, and neural network. The ML training and testing steps were as follows:

Step 1: All statistically significant metrics were imported to the classification learner app in MATLAB R2022a (MathWorks). Using the Feature Selection option, metrics were ranked using the analysis of variance method.
Step 2: Five predictor features with the greatest importance were selected as key predictors. Each feature predictor was used individually as a sole feature to train classification algorithms. Initially, all data were used to train and validate algorithms. Three algorithms with the greatest accuracy and largest area under the receiver operating characteristic curve (AUC) were selected for further analysis and training.
Step 3: In this step, one-third (1/3^rd^) of the data were randomly separated for manual testing and kept another two third (2/3^rd^) for training. Five sets of test and training data were applied to evaluate the accuracy of these three algorithms (3 × 5 = 15 tests). The average value of accuracy was used to compare the 3 algorithms' performance, and the algorithm with the largest mean accuracy was used as the main prediction algorithm.
Step 4: The best prediction algorithm was modified through hyperparameter optimization.
Step 5: Steps 2 to 4 were used for a combination of 2 to 7 predictor features.

## Results

### Automatic Parameter Extraction Pipeline Performance

**Figure 3:**
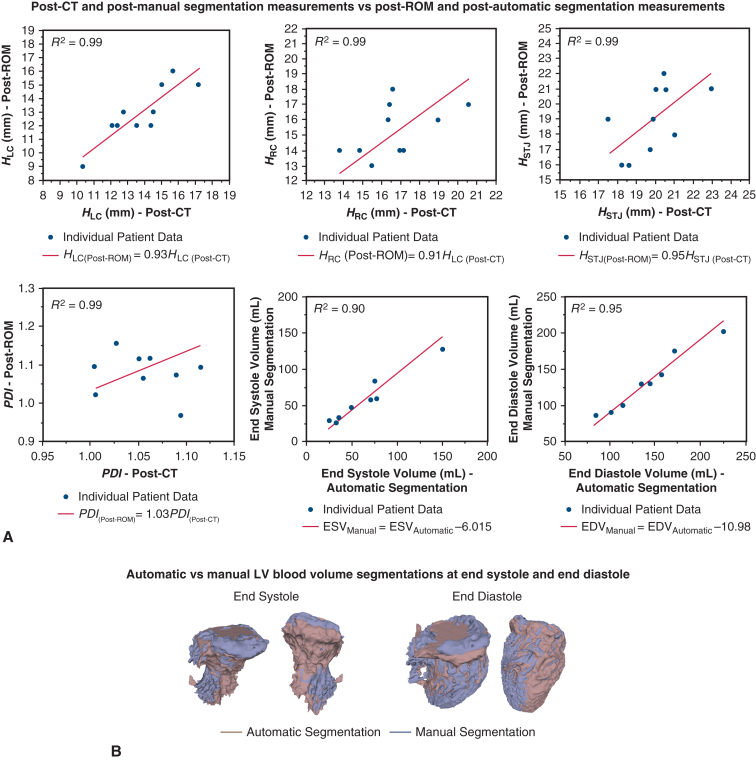
A, B, Comparison of geometric and hemodynamic measurements after manual methods versus measurements output from the computational pipeline. *CT*, Computed tomography; *ROM*, reduced order modeling; *H*_*LC*_, left coronary height; *H*_*RC*_, right coronary height; *H*_*STJ*_, sinotubular junction height; *PDI*, prosthesis deformation index; *ESV*, end-systolic index; *EDV*, end-diastolic index; *LV*, left ventricle.

The measurements obtained from computational pipeline were compared with the same measurements obtained directly using the post-TAVR CT or manual segmentation from pre-TAVR CT to evaluate the accuracy and clinical impact of the landmark-guided, intensity-based LV segmentation method (Figure 3, *A*). The difference in measured *H*_LC_ between post-ROM and post-TAVR CT ranged from 0.01 to 2.36 mm, *H*_RC_ difference was 0.22 to 3.57 mm, *H*_STJ_ difference varied between 0.42 and 3.06 mm, and PDI difference spanned from 0.009 to 0.12. Thus, the lowest mean percentage error (MPE) was 6.18% for PDI and greatest MPE was 10.62% for *H*_RC_ measurements (Table E2).

The difference in end systolic LV volumes between automatic and manual segmentation ranged from 2.06 to 22.02 mL (MPE of 6.15%), whereas end-diastolic LV volume difference was between 0.66 and 24.66 mL (MPE of 7.44%) (Figure 3, *A*, and Table E2). The automatic and manual segmentations of end-systolic and end-diastolic LV blood volumes values demonstrated close visual agreement between the meshes (Figure 3, *B*).

### Statistical Analysis and Development of HALT Prediction Metrics

**Figure 4:**
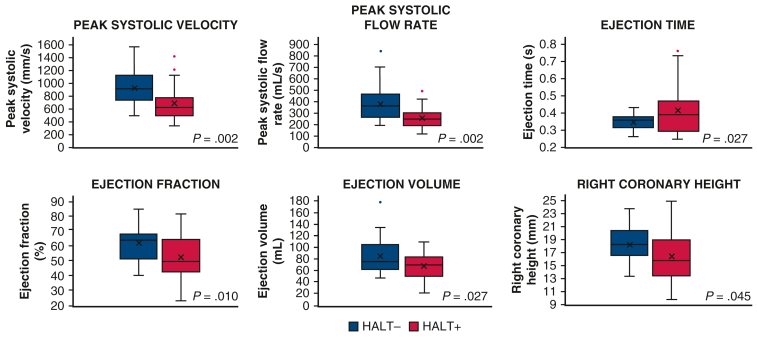
Boxplot distributions for statistically significant hemodynamic and geometric parameters. *HALT*, Hypoattenuated leaflet thickening.

The statistical analysis results for pre-TAVR hemodynamic measurements between HALT^−^ and HALT^+^ patients demonstrated that peak systolic blood flow rate and velocity through the TAV, ejection volume, and ejection fraction demonstrated statistically significant increase with HALT whereas ejection time demonstrated statistically significant decrease with HALT (Table E3). The right coronary height was the only geometric parameter that demonstrated statistically significant relationship with HALT occurrence (Table E4). The box-plot distributions for each of these statistically significant parameters are displayed in Figure 4. Other geometric parameters, such as *H*_NS_, *A*_stent_, and *H*_STJ_, demonstrated low *P* values and were therefore applied to develop nondimensional HALT prediction metrics (Table E4).

The metrics that were analyzed included the minimum circulation (*U*_min_) and normalized circulation (*NC*_min_) across all leaflets to account for the likelihood of HALT^+^ patients only having HALT in 1 or 2 leaflets. On the basis of the statistical performance of *U*_min_, a new quantity was developed to relate significant and independent geometric and hemodynamic parameters: *H*_RC_*, v*_sys_, and *t*_ej_. This metric was termed as the dimensional time rate of change in circulation $\left(\frac{\Delta U}{t}\right)$ (Table 1) and demonstrated greater significance than *U*_min_. $\frac{\Delta U}{t}$ was also normalized by using normalized *H*_RC_, *v*_sys_, and *t*_ej_ to yield ${\left(\frac{\Delta U}{t}\right)}_{norm}$, which showed further significance between HALT^+^ and HALT^−^ samples. Normalized metrics were developed to match the dimensionality of these shear stress and residence time (Table 1). Among these normalized metrics, *RT1* (*P* = .001), *RT2* (*P* = .002), and *SS1* (*P* = .013) displayed the most statistical significance of HALT risk.

**Table 1:** HALT prediction metrics constructed from primarily statistically significant post-TAVR geometric and hemodynamic parameters and their *P* values after 2-sample *t* tests

Metric	Definition	Expression	*P* value
*U*_min_	Minimum circulation across all leaflets	$\frac{A_{NS}*d*v_{sys}}{A_{S}}$	.0007
*NC*_min_	Minimum normalized circulation across all leaflets	$\frac{A_{NS}*d*v_{sys}*t_{ej}}{A_{S}*A_{C}}$	.032
$\frac{\Delta U}{t}$	Dimensional time rate of circulation change	$\frac{H_{RC}*v_{sys}}{t_{ej}}$	.00017
${\left(\frac{\Delta U}{t}\right)}_{norm}$	Normalized dimensional time rate of circulation change	$\frac{H_{RC,norm}*v_{sys,norm}}{t_{ej,norm}}$	.00011
${\left(\frac{\Delta U}{t}\right)}_{norm,\mathrm{mod}\;1}$	${\left(\frac{\Delta U}{t}\right)}_{norm}$ Modified 1	$\frac{H_{RC,norm}*v_{sys,norm}^{\frac{1}{3}}}{t_{ej,norm}}$	4.42 ∗ ^E−5^
${\left(\frac{\Delta U}{t}\right)}_{norm,\mathrm{mod}\;2}$	${\left(\frac{\Delta U}{t}\right)}_{norm}$ Modified 2	$\frac{H_{RC,norm}^{2}*v_{sys,norm}}{t_{ej,norm}}$	5.53 ∗ ^E−5^
*SS1*	Dimensional shear stress 1	$\frac{v_{sys}}{H_{NS,\min}}$	.013
*SS1*_norm_	Normalized dimensional shear stress 1	$\frac{v_{sys,norm}}{H_{NS,\min,norm}}$	.015
*SS2*	Dimensional shear stress 2	$\frac{Q_{sys}}{A_{stent,in}*H_{STJ}}$	.013
*RT1*	Dimensional residence time 1	$\frac{H_{NS,\max}}{v_{sys}}$	.001
*RT2*	Dimensional residence time 2	$\frac{ESV}{v_{sys}*A_{stent,out}}$	.002
*RT3*	Dimensional residence time 3	$\frac{V_{ej}}{v_{sys}*A_{stent,out}}$	.039

*HALT*, Hypoattenuated leaflet thickening; *TAVR*, transcatheter aortic valve implantation.

### ML Analysis of HALT Prediction Metrics

**Figure 5:**
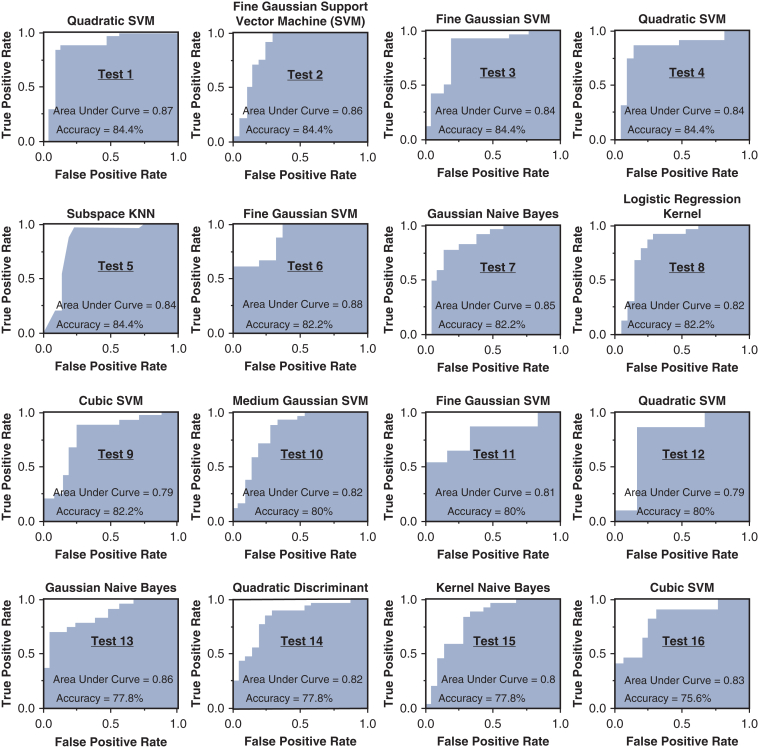
Receiver operating characteristic curves for all machine learning tests performed and the corresponding algorithms that maximized accuracies. Receiver operating characteristic curves are ordered from *left* to *right* by test number. *SVM*, Support vector machine.

**Figure E2:**
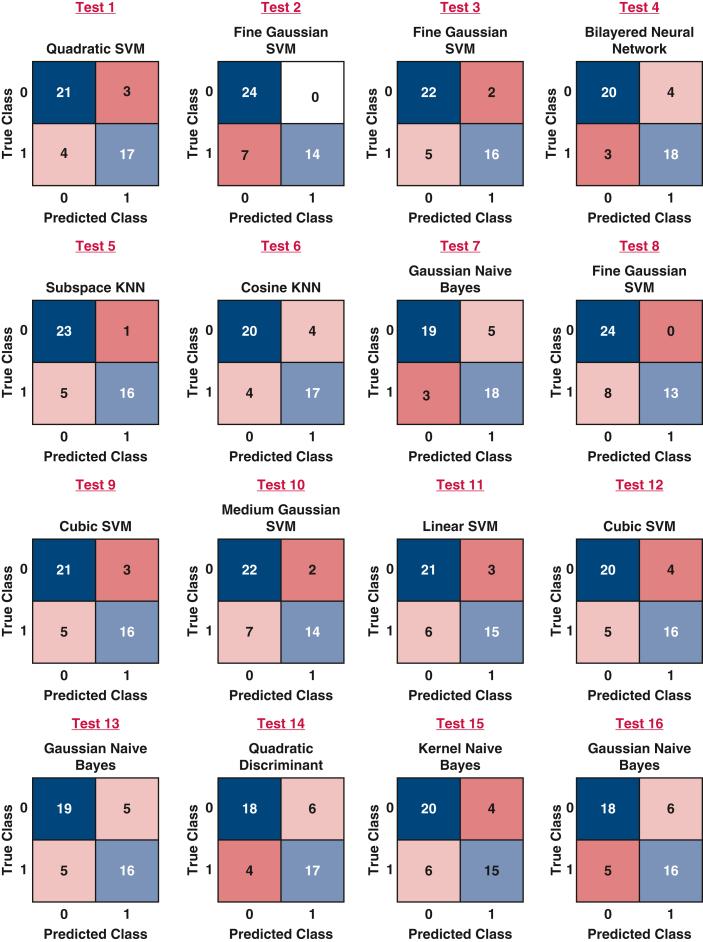
Confusion matrices for all machine learning tests performed and the corresponding algorithms that maximized accuracies. Matrices are ordered from *left* to *right* by test number. *SVM*, Support vector machine; *KNN*, K-nearest neighbors.

The identified statistically significant parameters and metrics in the previous section were imported as features for binary ML classification algorithms. The selected features and the resulting greatest accuracy and AUC for each ML test are presented in Table 2. The Confusion matrices for all tests and the algorithms used are displayed in Figure E2, and the receiver operating characteristic curves for all tests are illustrated in Figure 5. On the basis of results depicted in Table 2 and Figure 5, the combination of ${\left(\frac{\Delta U}{t}\right)}_{norm,\mathrm{mod}\;1}$, *U*_min_, and *SS1*_norm_ resulted in the greatest combined accuracy and AUC, with an accuracy of 84.4% and an AUC of 0.87.

**Table 2:** Best accuracy and AUC determined for each test consisting of 2 to 8 HALT prediction metrics as features

ML test	Feature	Accuracy	AUC
Test 1	${\left(\frac{\Delta U}{t}\right)}_{norm,\mathrm{mod}\;1}$	84.40%	0.87
	*U*_*min*_		
	*SS1*_*norm*_		
Test 2	${\left(\frac{\Delta U}{t}\right)}_{norm}$	84.40%	0.86
	*v*_*sys*_		
Test 3	${\left(\frac{\Delta U}{t}\right)}_{norm}$	84.40%	0.84
	*SS1*_*norm*_		
Test 4	${\left(\frac{\Delta U}{t}\right)}_{norm,\mathrm{mod}\;1}$	84.40%	0.84
	*SS1*_*norm*_		
Test 5	${\left(\frac{\Delta U}{t}\right)}_{norm}$	84.40%	0.84
	*v*_*sys, norm*_		
	*t*_*ej,norm*_		
	*H*_*RC,norm*_		
Test 6	${\left(\frac{\Delta U}{t}\right)}_{norm,\mathrm{mod}\;1}$	82.20%	0.88
	*NC*_*min*_		
	*SS1*_*norm*_		
Test 7	${\left(\frac{\Delta U}{t}\right)}_{norm,\mathrm{mod}\;1}$	82.20%	0.85
	*SS2*		
Test 8	${\left(\frac{\Delta U}{t}\right)}_{norm,\mathrm{mod}\;1}$	82.20%	0.82
	*v*_*sys, norm*_		
Test 9	${\left(\frac{\Delta U}{t}\right)}_{norm,\mathrm{mod}\;1}$	82.20%	0.79
	*U*_*min*_		
Test 10	*U*_*min*_	80.00%	0.82
	${\left(\frac{\Delta U}{t}\right)}_{norm}$		
	${\left(\frac{\Delta U}{t}\right)}_{norm,\mathrm{mod}\;2}$		
	*SS1*		
	*SS2*		
	*RT1*		
	*RT2*		
	*RT2*		
Test 11	${\left(\frac{\Delta U}{t}\right)}_{norm,\mathrm{mod}\;1}$	80.00%	0.81
	*RT2*		
Test 12	${\left(\frac{\Delta U}{t}\right)}_{norm,\mathrm{mod}\;2}$	80.00%	0.79
	*RT1*		
	*U*_*min*_		
	*SS2*		
Test 13	${\left(\frac{\Delta U}{t}\right)}_{norm,\mathrm{mod}\;1}$	77.80%	0.86
	${\left(\frac{\Delta U}{t}\right)}_{norm,\mathrm{mod}\;2}$		
Test 14	*U*_*min*_	77.80%	0.82
	${\left(\frac{\Delta U}{t}\right)}_{norm}$		
	${\left(\frac{\Delta U}{t}\right)}_{norm,\mathrm{mod}\;2}$		
	*SS1*		
	*SS2*		
	*RT1*		
	*RT2*		
Test 15	*U*_*min*_	77.80%	0.8
	${{\left(\frac{\Delta U}{t}\right)}_{norm}}^{2}*U_{\min}$		
	*RT3*		
	*SS2*		
Test 16	${\left(\frac{\Delta U}{t}\right)}_{norm,\mathrm{mod}\;2}$	75.60%	0.83
	*v*_*sys*_		

For metric definitions, see Table 1. *AUC*, Area under the receiver operating characteristic curve; *HALT*, hypoattenuated leaflet thickening; *v*_*sys*_, peak systolic velocity.

## Discussion

In this retrospective study examining the ability of pre-TAVR CT-based computational modeling in predicting post-TAVR geometric and hemodynamic parameters, we found that the resulting parameters demonstrated a high predictive accuracy for developing HALT after implantation. The findings in Table E3 support the hypotheses that lower flow rates and velocities would lead to decreased circulation in the neosinus regions, facilitating blood stasis and thrombus development, as discussed in previous works.^25^ Similarly, right coronary height demonstrating statistically significant relationship with HALT occurrence (Table E4), supports the hypothesis that greater coronary heights reduce neosinus washout, also facilitating blood stasis. Shear stress and residence time have been shown to characterize flow-mediated thrombogenic potential in the cardiovascular system and TAV leaflets.^27^, ^28^, ^29^ Therefore, normalized metrics were developed to match the dimensionality of these factors (Table 1).

**Figure E3:**
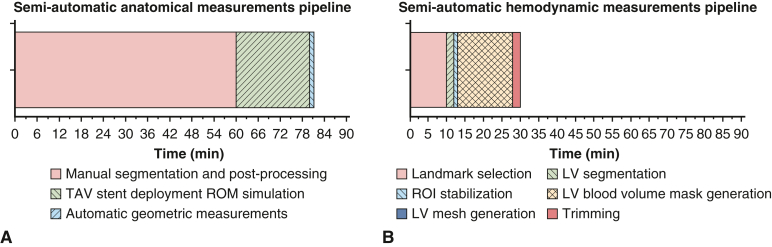
A, The semi-automatic anatomical measurements pipeline. B, The semi-automatic hemodynamic measurements pipeline. Approximate required time to complete each step of the computational pipeline. *TAV*, Transcatheter aortic valve implantation; *ROM*, reduced order modeling; *LV*, left ventricle; *ROI*, region of interest.

The semiautomatic computational pipeline developed in this study can predict these post-TAVR geometric and hemodynamic parameters in real-time fashion, within 2 hours per patient (Figure E3). The manual segmentation and postprocessing steps use proprietary software programs and require approximately 1 hour to train a human operator and 1 hour to segment per patient (Figure E3, *A*). This is followed by roughly 20 minutes to run a TAV stent deployment ROM simulation, compared with finite element analysis simulations, which can exceed 1 hour per patient. Finally, the automatic post-TAVR geometric measurements are conducted in less than 1 minute using open-source Python code. Therefore, this method has great potential to improve upon the time taken by CFD or FSI simulations.

A novel landmark-guided, intensity-based LV segmentation method further reduces procedure time and cost by using only open-source Python code to obtain hemodynamic measurements consistent with echocardiography results with minimal manual inputs and uncertainties within 30 minutes per patient (Figure E3, *B*). The automatic cardiac phase separation and manual CT landmark selection requires approximately 10 minutes to both train a human operator and run, after which, the remaining steps are automated. The ROI stabilization process across all phase images takes less than 1 minute. Then, the most time-consuming step is the LV blood volume mask generation, which requires 15 minutes on average depending on the complexity of the patient's LV anatomy. The final generation of the LV volumetric meshes can be completed in approximately 5 seconds per cardiac phase. Trimming of any excess volumes takes 2 minutes per phase depending on the interconnectivity between the ROI and excess regions.

For our ML analysis, various combinations of features were applied to train and test ML algorithms. We imported all possible features to MATLAB and ran analysis of variance Feature Selection test on them and selected the features with importance scores surpassing twice the average importance score of all features. At this point, several algorithms could provide similar accuracy, and the test data were automatically and randomly selected by MATLAB. Therefore, it was expected that repeating the training process on one dataset could provide us with variable accuracy and AUC. However, each classification algorithm depicted nearly consistent performance during each run, allowing 3 algorithms with highest average accuracy to be selected after 5 runs for further evaluation.

The number of features used for a test did not have a significant effect on the accuracy and AUC of that test (Table 2). Test 10, which had 8 features, ranked greater than or equal to tests having between 2 to 7 features but below others having between 2 to 4 features. In general, it can be noted that increasing the number of features used per test increases the overall accuracy, depending on the significance of each feature. However, the increased accuracy is mostly due to overfitting. In addition, using either metrics only or a combination of metrics and independent parameters as features did not significantly impact the accuracy and AUC of a test either. ${\left(\frac{\Delta U}{t}\right)}_{\text{norm}}$ is the best-performing HALT prediction metric that can be applied to any patient (Table 2 and Figure 5). Test 2 includes high accuracy and AUC, but it categorized most patients as HALT^−^. Tests 1 to 5 have the greatest accuracies, since they share the same accuracy, the tests with less input features are preferred; thus, tests 2, 3, and 4 would be the best choices. The AUC of Test 2 is larger than 3 and 4, but the incorrect predicted patients are distributed more uniformly in test 4. In test 4, we used ${\left(\frac{\Delta U}{t}\right)}_{\text{norm},\mathrm{mod}\;1}$ and *SS1*_norm_, which can be calculated with minimum effort and training by applying our automated method. Therefore, test 4 would be the authors’ choice for predicting the risk of HALT post TAVR. However, more patient data are required to train the ML algorithm and select the best-performing algorithm.

### Error Analysis

**Figure E4:**
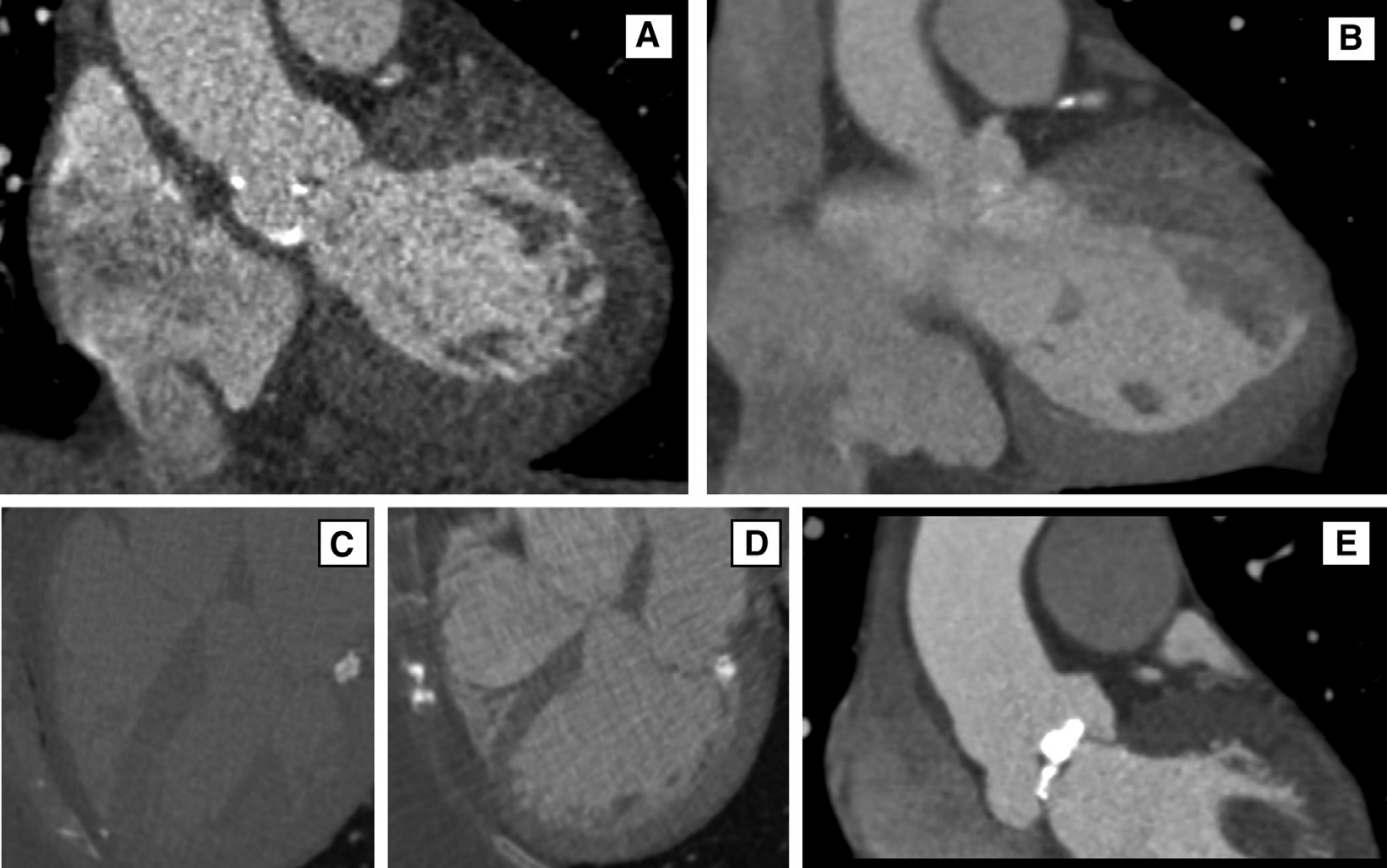
Sources of error from computed tomography images. A, Low image quality; B, presence of noise artifacts; C, low average pixel intensity for blood volume; D, close interconnectivity between LV and RV; and E, clipped left ventricle. *LV*, Left ventricle; *RV*, right ventricle.

Sources of error for the ROM-guided automatic post-TAVR geometric measurement method include low image quality (Figure E4, *A*), presence of noise (Figure E4, *B*), and other image artifacts. In addition, the manual segmentation of the aortic anatomies includes defining an intensity threshold for each geometric part (the root wall, calcium, and valve leaflets) by the user, and applying a uniform thickness to the segmented aortic wall, all of which could reduce accuracies of the 3-dimensional models and increase the degree of uncertainty. The accuracy of TAVR ROM model also impacts the overall error in autosegmentation and thrombus prediction model. This factor can be compounded by user variabilities in the manual selection of STJ plane landmarks from the post-ROM aortic root object and manual measurements of the coronary and STJ heights. When comparing these measurements to ones taken by clinicians from the post-TAVR CT, the post-CT image quality and user variability must also be considered.

The blood and cardiac tissues can be difficult to segment separately as the result of low average pixel intensity of the blood volume, which can lead to excess segmented volume that cannot be trimmed accurately (Figure E4, *C*). This was observed from a few CT images that were excluded from the dataset. In addition, the combination of similar pixel intensities between the LV and the right ventricle and close interconnectivity between them in the CT images can cause the marching cubes algorithm to “bleed into” the right ventricle regions for some or most of the moving images (Figure E4, *D*). Although the optional postprocessing step was intended to trim the excess volumes, it can be very time-consuming depending on the interconnectivity between target and excess regions, increasing the inaccuracy of the process. Finally, regions of the LV blood volume were also clipped in some of the CT images (Figure E4, *E*) and thus had to be excluded from the dataset.

### Limitations, Future Works, and Clinical Perspectives

This study was limited to a dataset of 45 consecutive patients from a cohort of more than 156 patients^24^ because of the specified anatomical and CT inclusion criteria. A larger cohort with more robust patient and procedural data will be applied in a future derivation and validation study. There was an over-representation of HALT (24 HALT^−^ and 21 HALT^+^) relative to the reported incidence^5^, ^6^, ^7^, ^8^ to limit class imbalance for ML analysis, potentially reducing the generalizability of current results to prospective clinical cases. Expanding the input dataset to include a larger, more representative patient cohort, with consideration of clinical and procedural factors, can allow the performance of sensitivity and specificity analyses to determine adaptive HALT^+^ and HALT^−^ thresholds for the most significant prediction metrics. Identification of these thresholds will allow the use of the pipeline on prospective TAVR cases or cases with blinded clinical outcomes to determine combinations of TAV size, degree of oversizing, depth of implantation, and prosthetic valve commissural alignments with the greatest risk of HALT development, which could augment procedural planning.

Further, the pre-TAVR and post-TAVR CT images for this dataset were acquired between February 2014 and March 2015, which meant that the images produced were lower quality than leading CT scanners available today. In addition, the dataset lacked standardized cusp-specific HALT scoring, which could provide a more nuanced understanding of how different predictors of HALT influence the location and severity of HALT formation. Our future work will integrate more nuanced scoring and characterization of HALT by applying established guidelines,^30^ increasing the precision compared with a binary scoring system.

The primary limitation of the computational pipeline is the inability to simulate patient-specific TAV leaflet motion to measure TAV leaflet positioning-dependent parameters, instead relying on in vitro experimental measurements. Currently, we are implementing a finite element analysis−based method to fit the TAV leaflets to stent frames deformed from patient-specific TAVR ROM simulations and pressurize them to mimic a cardiac cycle, followed by performing CFD simulations using the deformed TAV leaflets throughout the cardiac cycle. Even with the development of this one-way FSI model to simulate the interaction between blood flow and TAV leaflets, additional work will be needed to incorporate gradual thrombus development on TAV leaflets. In addition, our future work will also incorporate demographic, clinical, and post-TAVR echocardiographic features in the ML analysis that were previously found to be predictive of HALT, such as age, hypertension, hyperlipidemia, and mean aortic pressure gradient,^31^^,^^32^ alongside the current geometric and hemodynamic features.

In the future, with further refinements and testing, this pipeline could help in risk stratification, potentially augmenting monitoring and anticoagulation strategies. Indeed, although clinical studies have shown the lack of benefit of systematic anticoagulation after TAVR, it has been shown to reduce the rate of HALT. One can speculate that the absence of benefit might be explained by the fact that many patients never develop HALT and that systematic treatment might dilute the potential benefit that might be observed with targeted treatment. Thus, the ability to accurately detect patients at risk of HALT might be of significant clinical interest. Finally, all patients had tricuspid aortic valves and received a SAPIEN 3 TAV, so additional work is ongoing for the computational pipeline to expand the inclusion criteria to patients with bicuspid aortic valves and variable aortic anatomy, along with those receiving other TAVs, such as the EVOLUT self-expandable TAV (Medtronic). Indeed, it is conceivable that for a defined anatomy, the risk of HALT might be different from one valve platform to another, and the current approach could potentially be integrated into the decision-making process regarding device selection.

## Conclusions

In this work, a computational pipeline using pre-TAVR CT images of a patient as input and outputs post-TAVR geometric and hemodynamic measurements to predict risk of HALT was developed. The pipeline generates metrics statistically linked to thrombus risk, with an ability to predict HALT with more than 84% accuracy. After further validation of the model in a larger cohort, this tool may augment TAVR procedural planning and risk stratification.

## Conflict of Interest Statement

Dr Hatoum has filed a patent application on computational predictive modeling of thrombosis in heart valves. Dr Yeats has a patent pending as co-inventor of patents related to computational predictive modeling of heart valves and is a stakeholder in DASI Simulations. Dr Chen has patents pending on predictive computational modeling. Dr Neumann has received institutional grants from Boston Scientific, Biotronik, Medtronic, Edwards Lifesciences, and Abbott Vascular. Dr Blanke provides institutional CT core laboratory services to Edwards Lifesciences, Medtronic, Boston Scientiﬁc, Abbott Laboratories, Pi-Cardia, and Neovasc without direct personal reimbursement and is a consultant to Edwards Lifesciences. Dr Leipsic is a consultant to HeartFlow and Circle CVI, has stock options in HeartFlow, and provides institutional CT core laboratory services to Edwards Lifesciences, Abbott, Boston Scientific, and Medtronic. Dr Thourani is a consultant on research with Abbott Vascular, Boston Scientific, CryoLife, Edwards Lifesciences, Medtronic Corp, and Shockwave Medical and stakeholder in DASI Simulations. Dr Meier has received an institutional grant from Edwards Lifesciences. Dr Dasi is a stakeholder in DASI Simulations and has a patent pending as coinventor of patents related to computational predictive modeling of heart valves. Dr Sellers is a consultant to Edwards, Anteris Technologies, Excision Medical, and Medtronic; has received research support from Edwards, Medtronic, HeartFlow, Anteris Technologies, ViVitro Labs; and has stock options in Excision Medical and supported by a Michael Smith Health Research BC Scholar Award. All other authors reported no conflicts of interest.

The *Journal* policy requires editors and reviewers to disclose conflicts of interest and to decline handling or reviewing manuscripts for which they may have a conflict of interest. The editors and reviewers of this article have no conflicts of interest.

## Figures and Tables

**Table E1:** TAV size-specific parameters

TAV size, mm	*A*_S,int_, mm^2^	*d*_int_, mm	*A*_NS_, mm^2^	*w*, mm
20	20.94	2	35.63	3.83
23	27.70	2.3	40.98	4.40
26	35.40	2.6	46.33	4.97
29	44.03	2.9	51.67	5.55

*TAV*, Transcatheter aortic valve; *d*_*int*_, flow separated distance; *A*_*NS*_, stent flow separated area neosinus height; *w*, neosinus width.

**Table E2:** MAE and MPE for each geometric parameter following post-ROM and post-CT measurement methods

Parameter	MAE	MPE, %
*H*_LC_, mm	0.88	7.19
*H*_RC_, mm	1.41	10.62
*H*_STJ_, mm	1.23	9.13
*PDI*	0.03	6.18
*ESV*, mL	6.01	6.15
*EDV*, mL	10.98	7.44

*MAE*, Mean absolute error; *MPE*, mean percentage error; *ROM*, reduced order modeling; *CT*, computed tomography; *H*_*LC*_, left coronary height; *H*_*RC*_, right coronary height; *H*_*STJ*_, sinotubular junction height; *PDI*, prosthesis deformation index; *ESV*, end-systolic volume; *EDV*, end-diastolic volume.

**Table E3:** *P* values for hemodynamic parameters using HALT^−^ and HALT^+^ as the samples to perform 2-sample *t t*ests assuming unequal variances

Parameter	*P* value
*Q*_avg_	.021
*Q*_*sys*_	.0018
*Q*_*sys, norm*_	.0018
*v*_avg_	.031
*v*_sys_	.0017
*v*_sys, norm_	.0017
*t*_ej_	.027
*EF*	.010
*ESV*	.069
*EDV*	.477
*V*_ej_	.027

*HALT*, Hypoattenuated leaflet thickening; *Q*_*avg*_, average flow rate; *Q*_*sys*_, peak systolic flow rate; *Q*_*sys, norm*_, normal systolic flow rate; *v*_*avg*_, average systolic velocity; *v*_*sys*_, peak systolic velocity; *v*_*sys, norm*_, normal systolic velocity; *t*_*ej*_, ejection time; *EF*, ejection fraction; *ESV*, end-systolic value; *EDV*, end-diastolic value; *V*_*ej*_, ejection volume.

**Table E4:** *P* values for non-normalized and normalized post-TAVR geometric parameters after 2-sample *t tests* assuming unequal variances

Parameter	*P* value	*P* value after normalization
*H*_stent,min_	.395	.414
*H*_stent,max_	.397	.431
*H*_LC_	.296	.366
*H*_RC_	.045	.023
*H*_STJ,min_	.166	.178
*H*_STJ,max_	.363	.438
*PDI*	.483	N/A
*A*_S,ext_		
LCC	.358	.242
NCC	.187	.246
RCC	.282	.205
Minimum	.465	.494
Maximum	.319	.256
Sum	.398	.476
*d*_ext_		
LCC	.273	.295
NCC	.400	.311
RCC	.216	.180
Minimum	.341	.294
Maximum	.471	.427
*H*_NS_		
LCC	.199	.193
NCC	.415	.419
RCC	.170	.165
Minimum	.258	.256
Maximum	.258	.256
*V*_NS_		
LCC	.258	.355
NCC	.182	.237
RCC	.273	.117
Minimum	.279	.471

*TAVR*, Transcatheter aortic valve implantation; *H*_*stent,min*_, minimum stent height; *H*_*stent,max*_, maximum stent height; *H*_*LC*_, left coronary height; *H*_*RC*_, right coronary height; *H*_*STJ,min*_, sinotubular junction height minimum; *H*_*STJ,max*_, sinotubular junction height maximum; *PDI*, prosthesis deformation index; *N/A*, not available; *A*_*S,ext*_, inner wall flow separated area; *LCC*, left coronary cusp; *NCC*, non-coronary cusp; *RCC*, right coronary cusp; *d*_*ext*_, flow separated distance; *H*_*NS*_, neosinus height; *V*_*NS*_, neosinus volume.

## Supplementary Data

Video 1 Computational model deployment of balloon expandable TAV and post-TAVR geometric features measured. Video available at: https://www.jtcvs.org/article/S2950-6050(24)00041-X/fulltext.

## Appendix E1 Semi-Automated Left Ventricle (LV) Segmentation Method Description

### Pre-Transcatheter Aortic Valve Implantation Computed Tomography (CT) Phase Separation and Landmarks Selection

The recorded patient demographic information, heart rate, and cardiac phase percentage of each image series were extracted from pre-transcatheter aortic valve implantation CT images using RadiAnt DICOM Viewer (Medixant). A Python script was developed to automatically detect the phase percentage of each series and convert the Digital Imaging and Communications in Medicine images to a NumPy array. Eleven landmarks were manually selected for each patient to define the region of interest (ROI) containing the LV blood volume. From Figure 2, *A*, the MS1, MS2, and MS3 define the membranous septum plane to separate the LV from the right ventricle. AA1, AA2, and AA3 points in Figure 2, *A*, are the hinge points that mark the aortic annulus plane to mask out the aortic anatomies. MA1, MA2, and MA3 points (Figure 2, *A*) are the hinge points that specify the mitral annulus plane to mask out the left atrium anatomies, and the last 2 points, S1, and S2 (Figure 2, *A*) are arbitrary seed points within the LV blood volume domain to initiate region growing, which will be discussed later.

### Image Stabilization Across the Cardiac Cycle

The 3-dimensional coordinates of each landmark were recorded to define the ROI for image registration (Appendix E1).^E1^ This was used to map the points from CT image of 1 cardiac phase, defined as the fixed image, to the others, defined as moving images, to minimize the impact of LV movement throughout the cardiac cycle. SimpleITK's multiresolution registration framework was used, which consists of the transformation, selection of a similarity metric, optimization, and interpolation. The Euler 3D transformation was performed, which is a rigid 3D transform with rotation around a fixed center with translation. The following form of transformation was used:

$$\begin{array}{c} T\left(x\right)=A\left(x-c\right)+t+c\left(S1\right) \end{array}$$

where *A* is a matrix, point ***c*** is the geometrical center of each moving image passed as initial center of rotation, and vector ***t*** is the vector from the center of the fixed image to center of the moving image passed as the initial translation. A similarity metric was used in the registration to quantify how well the transform mapped the moving images on top of the fixed image. An algorithm that was designed to perform 3D positron emission tomography-CT registration in the chest using mutual information as a similarity criterion was used for the similarity metric.^E2^ After that, a gradient descent optimizer was implemented, which updates the current position at each iteration according to the following equation:

$$\begin{array}{c} p_{n+1}=p_{n}+learningRate\frac{\partial f\left(p_{n}\right)}{\partial p_{n}}\left(S2\right) \end{array}$$

The ROI of a moving image stabilized to the fixed image from a different cardiac phase (Figure 2, *B*).

### ROI Mask Generation and Detection of Pixel Intensity Thresholds

The fixed image and selected landmarks were used to generate a mask isolating the ROI from the remaining anatomies, which was applied to the moving images. The centers and normal vectors of membranous septum, aortic annulus, and mitral annulus planes (henceforth referred to as the boundary planes) were identified. They were used to determine whether each point along the CT image was located within the ROI or outside. The pixel intensity value was changed to 0 Hounsfield units for points located outside of ROI and kept the same for points within the ROI (Figure 2, *C*).

A histogram of pixel intensities greater than 0 Hounsfield units was plotted using the masked image. A characteristic intensity distribution (Figure 2, *D*) consists of 2 regions behaving similarly to bell curves, with the one encompassing lower-intensity values corresponding with LV tissue and the one to its right corresponding with the target blood volume. The minimum intensity threshold for the ROI (*Int*_min_) was determined by detecting the first local minimum of the histogram. However, since the histogram continues to trail after the second peak, the maximum intensity threshold (*Int*_max_) was approximated using the equation to follow:

(8) $$\begin{array}{c} {\mathrm{Int}}_{\max}={\mathrm{Int}}_{\min}+\frac{{Loc}_{\min}+{Loc}_{\max}}{2} \end{array}$$

where *Loc*_max_ is the second local maximum. The automatically detected intensity thresholds are highlighted in Figure 2, *D*.

### Reconstruction of LV Blood Volumes Across All Cardiac Phases

The intensities of the S1 and S2 points (Figure 2, *A*) across all cardiac phases were compared with the intensity thresholds to determine whether the value was outside the range. The pixels that were connected to each seed and lie within the intensity thresholds were labeled for segmentation across all images. Using a morphological closing image filter algorithm,^E2^ holes and tube-like structures smaller than 6 pixels ∗ 6 pixels ∗ 6 pixels in volume were removed for each phase segmentation. The Marching cubes algorithm developed by Lorensen and Cline^E3^ was applied to extract a polynomial mesh from 3D medical images and segment the LV in each cardiac phase. Then, the generated set of vertices and triangular faces were imported to Trimesh to create a triangular 3D mesh object for each phase. The 3D mesh objects were smoothed.^E4^ The final 3D geometries of the LV blood volume throughout a cardiac cycle are presented in Figure 2, *F*. The 3D geometries were saved as STL files and the volume of LV for each phase was automatically measured.
