# Supplementary figures and images for: A novel computational method to predict hypoattenuated leaflet thickening post-transcatheter aortic valve replacement using preprocedural computed tomography scans
Source: JTCVS Struct Endovasc. 2024 Dec 24;5:100041. doi: 10.1016/j.xjse.2024.100041 (PMC13244794; doi:10.1016/j.xjse.2024.100041)

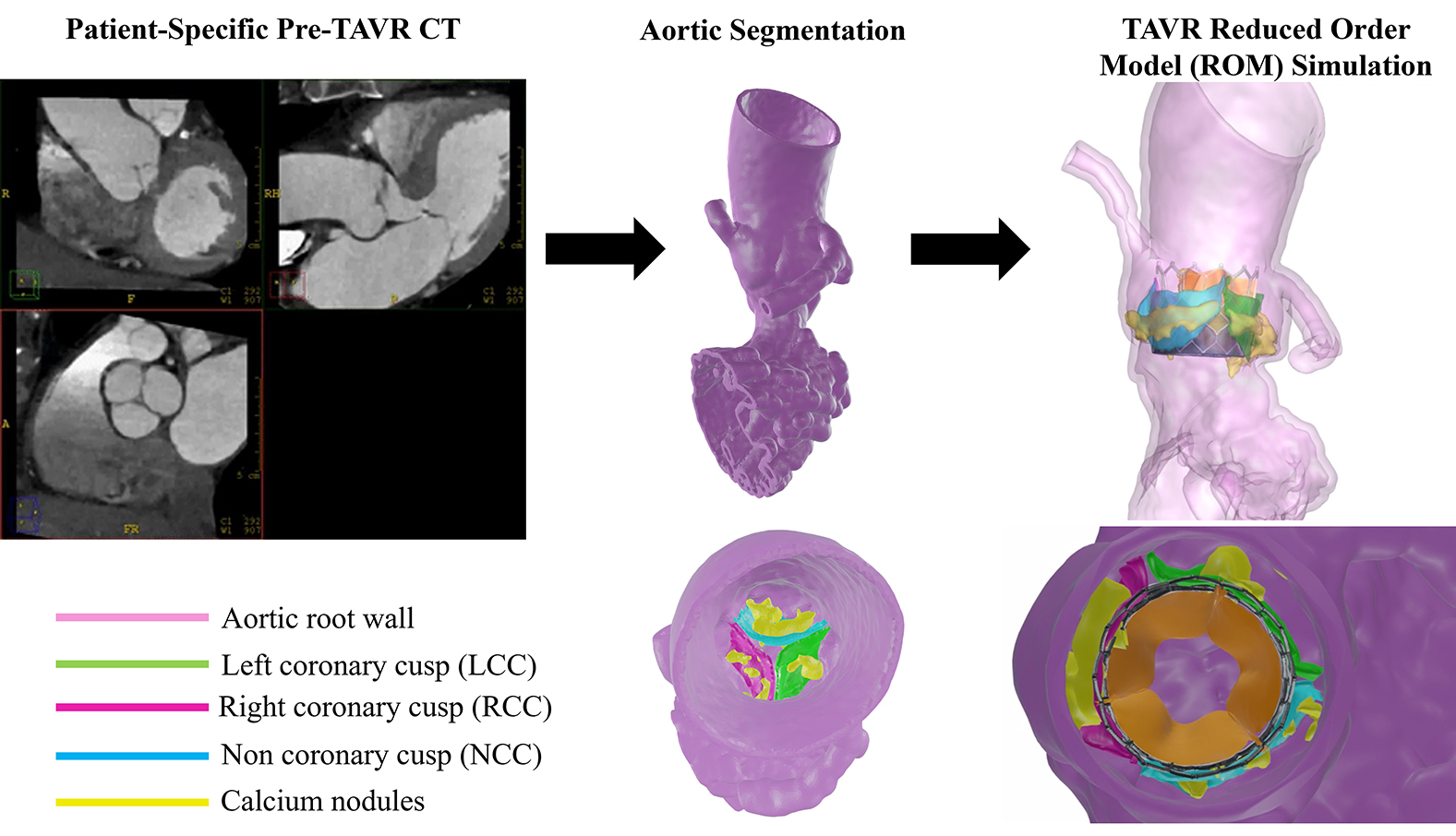

Supplement: Video 1 — Computational model deployment of balloon expandable TAV and post-TAVR geometric features measured. Video available at: https://www.jtcvs.org/article/S2950-6050(24)00041-X/fulltext. [file fx2.jpg]
